# Integrated Analysis of Chromatin and Transcriptomic Profiling Identifies PU.1 as a Core Regulatory Factor in Microglial Activation Induced by Chronic Cerebral Hypoperfusion

**DOI:** 10.1007/s12035-023-03734-9

**Published:** 2023-11-02

**Authors:** Zengyu Zhang, Pengpeng Jin, Zimin Guo, Zhilan Tu, Hualan Yang, Mengting Hu, Qinghua Li, Xingdang Liu, Weiwei Li, Shuangxing Hou

**Affiliations:** 1 Department of Neurology, Shanghai Pudong Hospital, Fudan University Pudong Medical Center, Shanghai, China; 2 Shanghai Medical College, Fudan University, Shanghai, China; 3 Department of Chronic Disease Management, Shanghai Pudong Hospital, Fudan University Pudong Medical Center, Shanghai, China; 4 Department of Nuclear Medicine, Shanghai Pudong Hospital, Fudan University Pudong Medical Center, Shanghai, China; 5 Institute of Pediatrics, Children’s Hospital of Fudan University, Fudan University, Shanghai, China

**Keywords:** Chronic cerebral hypoperfusion, Neuropathology, Microglia, IFN-β signaling, RNA-seq, ATAC-seq, PU.1

## Abstract

In addition to causing white matter lesions, chronic cerebral hypoperfusion (CCH) can also cause damage to gray matter, but the underlying molecular mechanisms remain largely unknown. In order to obtain a better understanding of the relationship between gene expression and transcriptional regulation alterations, novel upstream regulators could be identified using integration analysis of the transcriptome and epigenetic approaches. Here, a bilateral common carotid artery stenosis (BCAS) model was established for inducing CCH in mice. The spatial cognitive function of mice was evaluated, and changes in cortical microglia morphology were observed. RNA-sequencing (RNA-seq) and the assay for transposase-accessible chromatin with high-throughput sequencing (ATAC-seq) were performed on isolated mouse cortical brain tissue. Then, a systematic joint analysis of BCAS hypoperfusion-induced cortex-specific RNA-seq and ATAC-seq was conducted in order to assess the extent of the correlation between the two, and PU.1 was found to be greatly enriched through motif analysis and transcription factor annotation. Also, the core regulatory factor PU.1 induced by BCAS hypoperfusion was shown to be colocalized with microglia. Based on the above analysis, PU.1 plays a key regulatory role in microglial activation induced by CCH. And the transcriptome and epigenomic data presented in this study can help identify potential targets for future research exploring chronic hypoperfusion-induced brain injury.

**Supplementary Information:**

The online version contains supplementary material available at 10.1007/s12035-023-03734-9.

## Background

Vascular cognitive impairment (VCI) encompasses a wide range of cognitive dysfunctions caused by vascular conditions, including but not limited to subjective cognitive decline, mild cognitive impairment, and vascular dementia [1, 2]. As it is associated with a high rate of disability and mortality, there is an urgent need for effective prevention and treatment methods for this clinical problem [3, 4]. Pertinently, chronic cerebral hypoperfusion (CCH) is a significant contributing factor in the development of VCI [5, 6]. In addition to causing white matter lesions, decreased cerebral blood flow (CBF) can also cause damage to gray matter, including cortical and subcortical infarction, as well as disseminated lacunar or cerebral watershed infarction [7, 8]. It is probable that prolonged cerebral hypoperfusion and reduced CBF can result in an increase in the structural impairment of neurons, accompanied by neuronal loss, brain atrophy, and other symptoms, ultimately leading to lasting brain tissue damage and cognitive dysfunction [9].

From a pathophysiological perspective, the mechanisms involved in CCH are complex, for reasons including that they involve neuroinflammation, oxidative damage, apoptosis, endothelial dysfunction, and disruption of the blood-brain barrier. Currently, immuno-inflammatory injury is widely considered to be one of the most important pathological mechanisms that contribute to CCH [10]. During a prolonged period of chronic ischemia, the brain’s primary immune cells, microglia, can develop a highly pro-oxidative phenotype which promotes the transition from acute to chronic inflammation in the brain [11]. This leads to the production of cytotoxic factors and inflammatory mediators, such as TNF-α, IL-1β, and NO, which further exacerbate neuroinflammation and neuronal death [12, 13]. Additionally, in cases of ischemic brain injuries, microglia can determine the fate of cell populations in the central nervous system through cell-to-cell interactions, in which they act as hubs of intercellular communication. Therefore, the focus of our research is on the effects of CCH on microglia.

However, the underlying molecular mechanisms behind brain damage and microglial morphological alterations induced by CCH remain insufficiently understood. Several previous studies have reported the activation of the type I interferon (IFN-I) signaling pathway in rodent models of brain injury, where the interferon-beta (IFN-β) signaling and response can promote the release of pro-inflammatory cytokines and modulation of brain injury, involving interactions between different cell types in the brain [14]. In our previous work, RNA sequencing (RNA-seq) was performed to identify cortex-specific gene expression changes caused by BCAS hypoperfusion [15]. It was found that neuroimmune response mediated by IFN-β signaling plays an important role in the regulation of brain injury induced by CCH. However, it is unclear whether or not IFN-β signaling is closely related to the changes in microglial phenotypes induced by CCH. RNA-seq is mainly used for difference analysis and biological function enrichment [16]. Gene expression involves a very complex process and is regulated by transcriptional factors, coregulatory factors, and epigenetic modifiers. Epigenetic changes also play an important role in disease progression [17]. Analysis of the epigenome is conducted using free nuclei obtained in the tissue, and the epigenome can be evaluated for more than 95% of the non-protein coding regions of the genome. The non-coding region contains millions of candidate cis-acting elements that affect gene expression, including promoters and enhancers. Just as genes are the basic unit of transcriptome analysis, these candidate cis-acting elements are the basis of epigenetic analysis and can be combined with transcription factors (TFs) to cause nucleosome displacement and form accessible chromatin regions. Also, the assay for transposase-accessible chromatin with high-throughput sequencing (ATAC-seq) is used to study chromatin accessibility [18]. As chromatin plays a critical role in regulating gene expression, and gene regulation is a fundamental process in the development and progression of disease, combined analysis of the chromatin and genes can further reveal the potential pathologies and provide new ideas for the treatment of CCH.

Based on our results, as well as those of previous studies [15], we successfully established the 0.16/0.18 mm BCAS hypoperfusion model for gray matter lesions, with microglia manifesting a state of neuroinflammatory activation in the cerebral cortex. We then utilized both RNA-seq and ATAC-seq to study the effect of chromatin accessibility on gene expression as a whole, in order to investigate the molecular mechanics of CCH. This study aims to verify the cellular and molecular changes that occur with cortical injury and microglial activation caused by CCH, and to further explore the upstream regulatory mechanics. It can serve as a theoretical foundation for the study of prospective treatment targets for hypoperfusion-induced brain injury.

## Methods

### Animals

The animal experiments in this study were in compliance with the ARRIVE guidelines [19]. Ten-week-old male C57BL/6 mice were provided by Beijing Vital River Laboratory Animal Technology. Mice were housed in cages on a 12 h light/dark cycle with free access to food and water. After one-week acclimation, the mice were divided into 2 groups by random number method: the BCAS group and the sham group. A total of 89 mice were used in this study; 12 mice failed to survive the BCAS operation. The remaining 77 mice were used for the entire experiment. Total 19 mice were used in the Morris water maze test, 30 mice in the 3-week pathological staining, 10 mice in the western blot analysis, 8 mice in the ATAC-seq, and 10 mice in the qRT-PCR experiment. Approval was granted by the Institutional Animal Care and Use Committee of Shanghai Pudong Hospital, Fudan University Pudong Medical Center (No. WZ-07, 2023).

### Surgical Procedures for Bilateral Common Carotid Artery Stenosis

BCAS surgery was performed as previously described [20]. Briefly, we induced the BCAS model using microcoils made of piano wire with inner diameters of 0.18 mm and 0.16mm (Xi’an anruike Biotechnology Co., Ltd.). For the BCAS surgical procedure, mice aging 11 weeks were anesthetized with 3% isoflurane, then maintained with 1.5–2% isoflurane in 70% N_2_ and 30% O_2_. Mice in the BCAS group were exposed the bilateral common carotid arteries (CCA) and detached the vagus nerve. Then, the 0.16 mm microcoil was first rotated around the CCA below the carotid bifurcation on the right side. And the 0.18 mm microcoil was wound on the same position on the left side of the CCA about one hour later. For sham-operated mice, the CAA was exposed without applying the microcoils. After surgery, mice were warmed with a heating pad at 37°C to maintain body temperature.

### Morris Water Maze Test

Morris water maze (MWM) test was performed as described previously with some modifications [21]. A 120 cm pool filled with white-colored water and a 9 cm platform were used for the whole experiment period. Prior to training, mice were placed in the pool and assessed for their ability to find and climb onto the visible platform within 60 s. Mice that failed to accomplish the task at least twice were excluded from further testing. The platform was then relocated and lowered into a fresh pool of colored water, where it was buried 1 cm beneath the surface. Mice underwent a 5-day training period, with 4 trials per day and 60 s per trial, to locate the hidden platform. To guarantee that the mice used their visual-spatial memory to locate the hidden platform, four start positions were used. On the sixth day, the platform was taken out, and mice were dropped into the pool at a fresh start location. Time spent in the platform area and the target quadrant and the number of platform crossings within 120 s were recorded.

### Preparation of Paraffin Sections and Nissl Staining

The brains were removed after perfusion of the animals and fixed in freshly prepared 10% formalin for 24 hours. Then the brain tissues were dehydrated and embedded in paraffin according to the routine procedure. And the paraffin blocks were cut into 4-μm-thick sections using a microtome. For Nissl staining, the steps were as follows: First, the paraffin embedded slides were deparaffinized and rehydrated. Then, the brain sections were stained with crystal violet at room temperature for 20 min and differentiated in differentiation solution for 5 s. At last, the slides were dealt with anhydrous ethanol and xylene solution respectively and sealed with neutral gum.

### Preparation of Frozen Sections

The experimental animals were anesthetized by intraperitoneal injection of pentobarbital (150 mg/kg) and then perfused transcardially with cold phosphate-buffered saline (PBS), followed by 4% paraformaldehyde (PFA). Brains were removed, post-fixed in 4% PFA for 12 h, and submerged in 15% sucrose in PBS for 24 hours, then 30% sucrose in PBS for 48 hours. Coronal sections of 30 μm were made using a freezing microtome (CM 1900, Leica, Germany). All prepared slices were transferred to the cryoprotection solution until processing.

### Hematoxylin Staining

Hematoxylin staining was performed on the sections using the Solarbio kit (G1120) with minor modification. For the staining procedure, brain sections were fixed in methanol and stained with hematoxylin. Excess background staining was removed by a quick rinse in 1% HCl in alcohol, followed by treatment with the differentiation solution. At last, slides were passed through 70% alcohol and anhydrous alcohol, and mounted with xylene mountant. Images were taken by the Olympus slide scanner (Olympus Slideview VS200). The scan was a whole brain acquisition, with 5 brain slices from 10 animals in each group. View of the left or right hemisphere was selected. “Measures a freehand polygon” in the OlyVIA (Olympus) software was used to manually define the infarct area. Then the software automatically calculated the area values inside the selection. Cerebral infarction area was calculated by dividing infarct area of the ipsilateral hemisphere by total hemisphere.

### Immunofluorescence Staining

Brain slices were washed in 1x PBS solution and then blocked with 5% goat serum (Thermo Fisher, 16210064) in 1% Triton X-100 in PBS for 1 hour. Subsequently, the free-floating sections were incubated with rabbit anti-IBA1 (ab178846, Abcam), rat anti-CD68 (ab53444, Abcam), rabbit anti-GBP2 (Abclonal, A12994), rabbit anti-PU.1 (2258S, CST), goat anti-IBA1 (ab5076, Abcam), and overnight at 4 °C. These sections were then washed in 1% Triton X-100 in PBS and treated with secondary antibodies (Donkey anti-rabbit Alexa Fluor 488, Invitrogen, A-21206; Donkey anti-goat Alexa Fluor 594, Jackson, 705-585-003; Donkey anti-rat Alexa Fluor 594, Jackson, 712-585-153) for 1 hour at room temperature. The nuclei of cells were stained with DAPI (Thermo Fisher, 62248, 1: 10000 dilution) for 15 min. Sections were observed under an Olympus slide scanner (Olympus Slideview VS200). High-resolution images were captured from the confocal microscopy (SP8 LSCM, Leica, Germany). Field of view of the cerebral cortex was observed. Images were processed using the ImageJ software. 3D reconstructions were performed in Imaris software. Quantitative analysis of process length of microglial cell was conducted. Sholl analysis was used to evaluate cell morphology.

### Western Blotting

The right cerebral cortex tissues of mice from both groups were removed and lysed in RIPA lysis buffer supplemented with protease/phosphatase inhibitor cocktail (Beyotime-P1005). The concentration of each protein sample was measured by bicinchoninic acid (BCA) assay. Equivalent amount of protein was denatured with 5x loading buffer at 95°C for 10 min and stored at −80°C until further analysis. Equal amounts of protein were loaded on SDS-polyacrylamide gel electrophoresis gels and transferred onto polyvinylidene difluoride membranes and transferred to PVDF membranes (Millipore, Billerica, MA, USA). Then the membranes were blocked with 5% BSA in 1%TBST solution and incubated with primary antibody in the blocking solution overnight at 4°C. Primary antibodies used included anti-NeuN (Abcam, ab177487), anti-Iba1 (Abcam, ab178846), anti-GAPDH (Abclonal, AC001), and anti-β-actin (Abclonal, AC026) antibodies. Horseradish peroxidase-conjugated secondary antibody (Goat anti-rabbit, Beyotime, A0208) was added for 1 hour at room temperature in the blocking solution. The protein signals were detected with chemiluminescence imaging. Blots were processed using the ImageJ software, and the intensities of GAPDH or β-actin were used to normalize the quantities of target proteins.

### ATAC-seq Library Preparation and Sequencing

ATAC-seq was conducted using freshly isolated cerebral cortex from each group. In brief, isolated cortex was homogenized in the lysis buffer (0.32 M sucrose, 3 mM MgA_ce2_, 5 mM CaCl_2_, 10 mM Tris-HCl, pH=8, 0.1 mM EDTA, 0.1% NP-40), followed by washing in a sucrose buffer (10 mM Tris-HCl, 3 mM MgA_ce2_, 1.8 M sucrose). And nuclei were collected by centrifugation at 2000 rcf for 5 min. Then ATAC-seq was performed by following published protocol with some modifications [22]. In brief, the cell pellets (100 k nuclei) were incubated with 5 μl of Tn5 enzyme in 50 μl of transposition mix (TD501, TruePrep DNA Library Prep Kit V2 for Illumina, Vazyme, China) at 37 °C for 30 min. Then, DNA was purified using a Minelute Gel Extraction Kit (Qiagen, 28604) and eluted in 27 μl of EB buffer. The PCR reaction was set up as follows: 24 μl of DNA, 10 μl of 5 × TAB, 5 μl of PPM, 5 μl of N5 primer, 5 μl of N7 primer, and 1 μl of TAE buffer, and the PCR was performed applying the following cycling conditions: 72 °C for 3 min, 98 °C for 30 s, then 12–13 cycles of “98 °C for 30 s, 60 °C for 30 s, and 72 °C for 30 s,” followed by a final 72 °C extension for 5 min. Amplification products were further purified with a VAHTS® DNA Clean Beads Kit (N411, Vazyme, China). The quality of the library was evaluated using the Agilent 2100 TapeStation System (Agilent Technologies, Inc., Santa Clara, CA, USA), and the concentration was measured with Qubit4. The libraries were pooled and subjected to paired-end sequencing with 150-bp reads length on Illumina Nova-Seq 6000 platform (Illumina, San Diego, CA, USA).

### Extraction of mRNA and Quantitative Real-time PCR (qRT-PCR)

The TRIzol method (Invitrogen) was applied to extract total RNA from the right cortex tissues followed by removal of genomic DNA contamination using RNAse-free DNAse (Invitrogen). RNA concentration and purity were analyzed via NanoDrop 2000 (Thermo Fisher Scientific, Wilmington, DE). Then, total RNA was reverse-transcribed using Evo M-MLV RT Kit (AG11728, Accurate Biology). And qRT-PCR (Thermo Fisher Scientifc Applied Biosystems QuantStudio5) was then performed using SYBR® Green Premix qPCR Kit (AG11718, Accurate Biology). Primer sequences used in this experiment are provided in Supplementary Table 1. Data were normalized using *Gapdh* as the reference housekeeping gene. Relative expression levels were calculated using the 2^−∆∆CT^ method.

## Data Analysis

### ATAC-seq

Raw data was obtained in FASTQ format, and FastQC was used for quality control analysis (see Supplementary Table 2). And data preprocessing was conducted to obtain high quality clean data. Then, paired-end clean data was aligned to the reference genome (*M. musculus*, UCSC mm10) using Bowtie2 (v2.3.4.3) [23]. Samtools (v1.9) was used to convert, sort, and build the alignment files index after removing duplications[18]. MACS2 callpeak function was used for peak calling [24], and differential change peaks were identified using the R package DiffBind (see Supplementary Table 3) [25]. Annotation of peaks was performed using R packages “ChIPseeker,” “clusterProfiler,” “org.Mm.eg.db,” and “TxDb.Mmusculus.UCSC.mm10.knownGene” [18]. Motif search was conducted using the “fndmotifs.pl” function of HOMER, and the TFs were then annotated.

### Integrative Analysis of Bulk RNA-seq and Published scRNA-seq

Analysis was performed as described previously [26]. In brief, cell clusters were generated with Seurat in the UMAP plot using the published scRNA-seq dataset (GSE60361) [27]. For our cortex-specific RNA-seq dataset (GSE210666), differentially expressed genes (DEGs) analysis was performed using DESeq2 (DEGs obtained by RNA-seq are presented in Supplementary Table 4). Then, cell-type enrichment analysis was conducted by using DEGs from our RNA-seq dataset and the above scRNA-seq dataset. The identified DEGs were divided into two groups: BCAS-induced up-regulated genes (Up_genes) and BCAS-induced down-regulated genes (Down_genes). In addition, Seurat’s DoHeatmap visualizes the expression value of scRNA-seq dataset for the individual gene in the two groups. Notably, only those genes detected in scRNA-seq were shown. Moreover, the Seurat function FindAllMarkers was used to identify marker genes of each cell cluster in scRNA-seq dataset with the parameters by default. Enrichment scores of genes in these two groups in each cell cluster were generated using R package GeneOverlap. Furthermore, cell-type deconvolution analysis was calculated using the *UCell* algorithm from the open-source R package at https://github.com/chuiqin/irGSEA.

### Integrative Analysis of RNA-seq and ATAC-seq

For our cortex-specific ATAC-seq dataset (GSE215893), significant differential change peaks that are located on gene promoters were extracted, and peak-related genes were generated. Then, Venn diagram was drawn using the VennDiagram R package. And the shared up- and down-regulated genes of both RNA-seq and ATAC-seq were identified (Supplementary Table 5). In addition, Gene Ontology (GO) enrichment analysis was conducted by using ShinyGO v0.77 (http://bioinformatics.sdstate.edu/go/). Then, we conducted cell type enrichment analysis on the shared DEGs using the cortex-specific single-nuclei RNA sequencing (snRNA-seq) dataset (GSE229259). Single nuclei colored by cell types in all combined samples (combined, sham, and BCAS groups) were displayed using t-distributed stochastic neighbor embedding (t-SNE) plot. And the enrichment of snRNA-seq for shared “Up_genes” and “Down_genes” was performed using irGSEA analysis with the *UCell* algorithm.

### Statistical Analysis

All experiments and data analysis were conducted under investigator-blinded conditions. Statistical analyses were performed using GraphPad Prism Software. Paired *t* test (two-tailed) was used for the data comparisons of 0.18 mm side and 0.16 mm side in BCAS group samples, while an unpaired *t* test (two-tailed) was performed in the comparisons made between sham and BCAS groups. When more than two groups were compared, one-way analysis of variance (ANOVA) and two-way ANOVA were used to test inter-group differences. All data are expressed as mean ± SEM, and *p* < 0.05 was considered statistically significant.

## Results

### Behavioral Alterations after BCAS Hypoperfusion

**Fig. 1:**
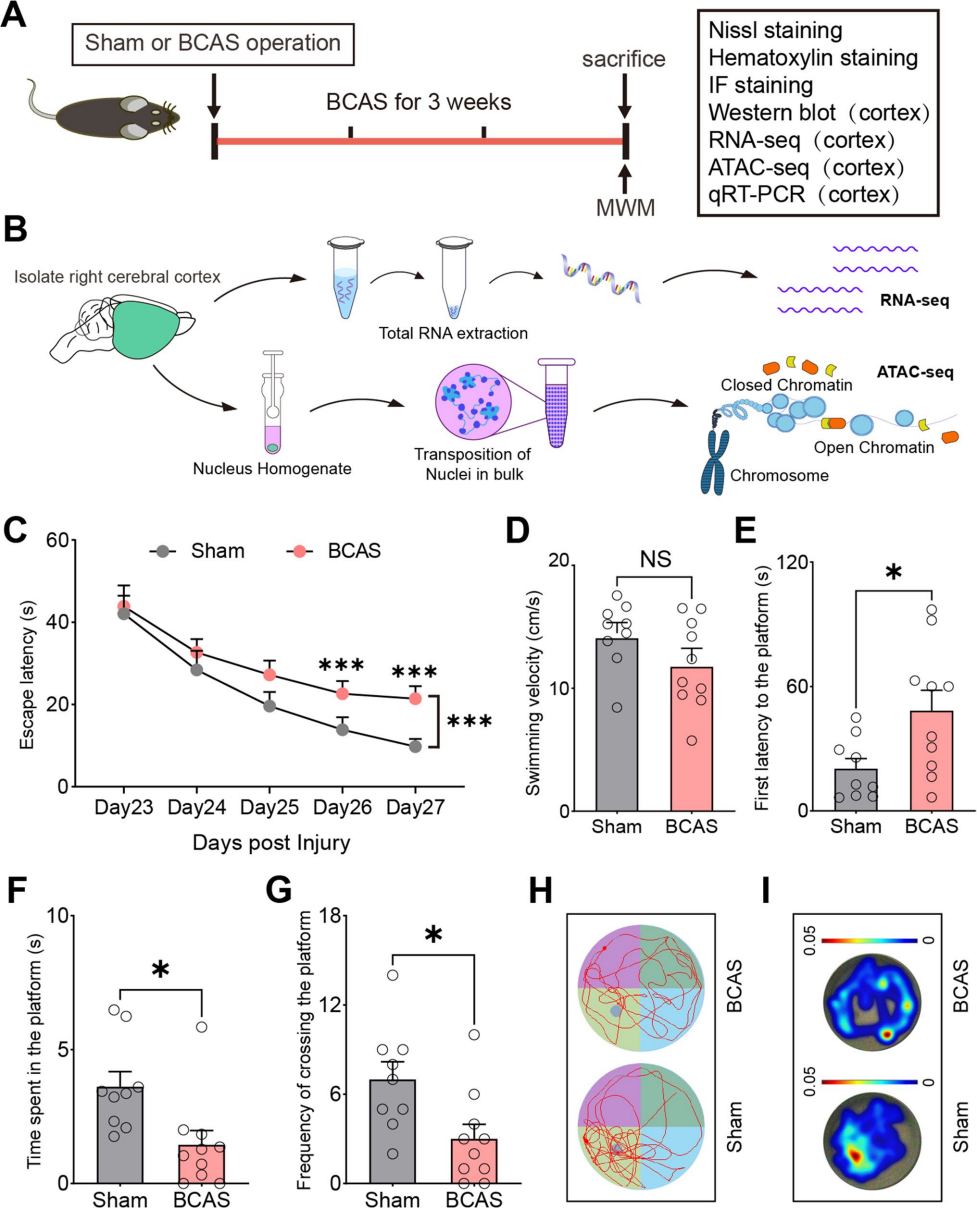
Changes of behavioral phenotype after BCAS hypoperfusion. **A** Experimental design. BCAS bilateral carotid artery stenosis, MWM Morris water maze test, IF immunofluorescence staining. **B** Flow chart of the cortex-specific RNA-seq and ATAC-seq experiments. **C** Escape latency of sham and BCAS mice in MWM test (learning phase). Subgroups, *F*_(1, 85)_ = 82.77, *p* < 0.001; time, *F*_(4, 85)_ = 173.3, *p* < 0.001; subgroups × time, *F*_(4, 85)_ = 5.319, *p* < 0.001. Two-way ANOVA test. **D–G** Swimming velocity, first latency to the platform, time spent in the platform (%), and frequency of crossing the platform of sham and BCAS mice in MWM probe test. Unpaired *t* test (two-tailed). **H–I** Representative track plots and heatmaps of sham and BCAS mice in MWM probe test. Data are presented as means ± SEM. *N* = 9–10 mice per group. NS = not significant, *p < 0.05, ***p < 0.001

Figure 1A–B shows the overall experiment scheme. In order to evaluate functional changes after prolonged cerebral hypoperfusion in the current BCAS model, we utilized a Morris water maze to test spatial cognitive function between the two groups. The results indicated that mice in the BCAS group showed impaired spatial learning ability compared with the mice in the sham group postoperatively (Fig. 1C). However, BCAS mice did not show motor dysfunction, as there was no difference in swimming speed between the two groups during the learning phase (Fig. 1D). The BCAS mice also showed significant reduction in latency to first entry in the platform zone and spent significantly less time than the sham mice in the target platform, as well as showed a reduction in the number of successful platform crossings in the probe trial (Fig. 1E–G). These results indicated that mice with BCAS hypoperfusion resulted in learning deficits and reference memory loss 3 weeks post operation.

### BCAS Hypoperfusion-induced Cortical Lesions and Phenotypic Changes of Microglia

**Fig. 2:**
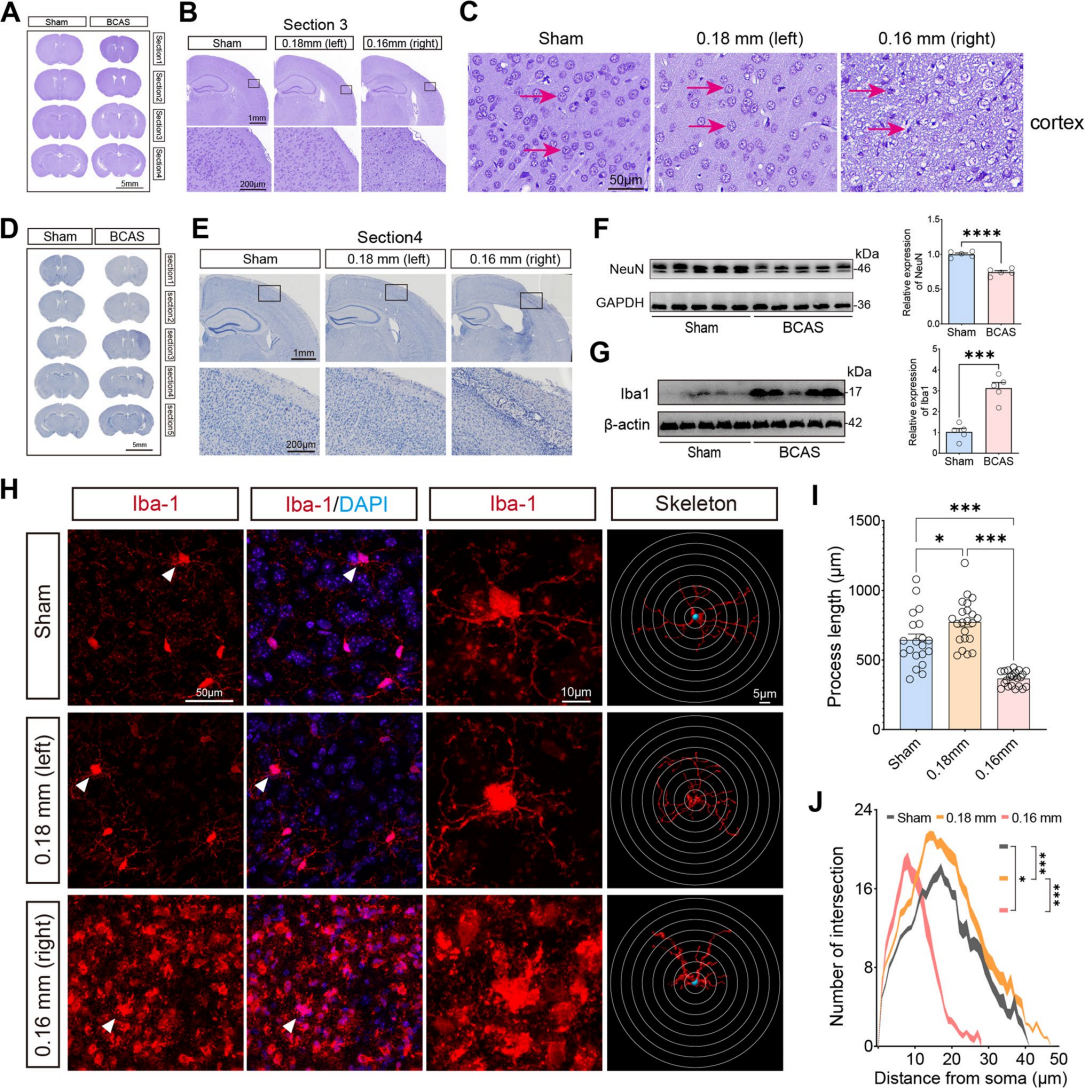
Cortical lesions and phenotypic changes of microglia caused by BCAS hypoperfusion. **A** Representative pictures of Nissl staining in sham and BCAS mice 3 weeks postsurgery. **B** BCAS hypoperfusion induced neuronal damage of cerebral cortex on the 0.16 mm stenosis side. **C** Loss of neurons on the 0.16 mm stenosis side of BCAS mice in a high-power field. **D** Consecutive coronal brain slices stained with hematoxylin following BCAS hypoperfusion. **E** Representative pictures depicting the pathological changes of the cerebral cortex. **F** Representative western blot images of NeuN in the cerebral cortex are shown on the left, and the statistical analysis of relative protein expression level is on the right. Unpaired *t* test. *N* = 5/group. **G** Representative western blot images of Iba1 in the cerebral cortex are shown on the left, and the statistical analysis of relative protein expression level is on the right. Unpaired *t* test. *N* = 5/group. **H** Representative images of anti-Iba1 immunofluorescence staining in cortical region from sham and 0.16/0.18 mm BCAS group. **I** Bar plot quantifies the length of the microglia process. One-way ANOVA test. *N* = 20–22 processes/5 animals/group. **J** Sholl analysis for microglia branch complexity. Two-way ANOVA test. *N* = 20–22 processes/5 animals/group. Data are expressed as mean ± SEM. *p < 0.05, ***p < 0.001, ****p < 0.0001

We first evaluated the histological changes after BCAS hypoperfusion via Nissl staining and hematoxylin staining of different sections. On the 0.16 mm stenosis side, significant neuronal loss was evident in the region of cerebral cortex (Fig. 2A–C). The entire hemisphere ipsilateral was atrophied and showed thinning of the cerebral cortex due to 0.16 mm stenosis (Fig. 2D–E). We then compared the mean percent infarction size in each section. The results showed significantly larger infarction on the 0.16 mm side compared to the 0.18 mm side (Supplementary Fig. 1). In addition, western blot results showed that BCAS hypoperfusion increased the expression of Iba-1 and decreased the expression of NeuN in the cortical region (Fig. 2F–G). Thus, it can be concluded that BCAS hypoperfusion resulting from 0.16 mm stenosis can cause clearly evident histological injury. In our previous work, we found activation of microglia occurred mostly in areas of neuronal injury, including the cerebral cortex on the 0.16 mm stenosis side of BCAS mice [15]. Microglia, as the primary innate immune cells and important cellular mediators in the brain, play an important role in neuroinflammation. Therefore, we performed anti-Iba1 staining to examine the condition of microglia, focusing on the cerebral cortex (Fig. 1H). Chronic hypoperfusion has been reported to lead to microglia activation, often described as swollen cell bodies with thicker and shorter processes or amoeboid-like morphology [15]. In our current BCAS hypoperfusion mouse model, it was found that microglia on the 0.18 mm side of BCAS group exhibited hyper-ramification morphology characterized by an increased length of branches (Fig. 1I) and an increased number of branch intersections (Fig. 1J), while microglia on the 0.16 mm side of BCAS group displayed amoeboid-like morphology featured by decreased branch length (Fig. 1I) and decreased number of branch intersections (Fig. 1J).

**Fig. 3:**
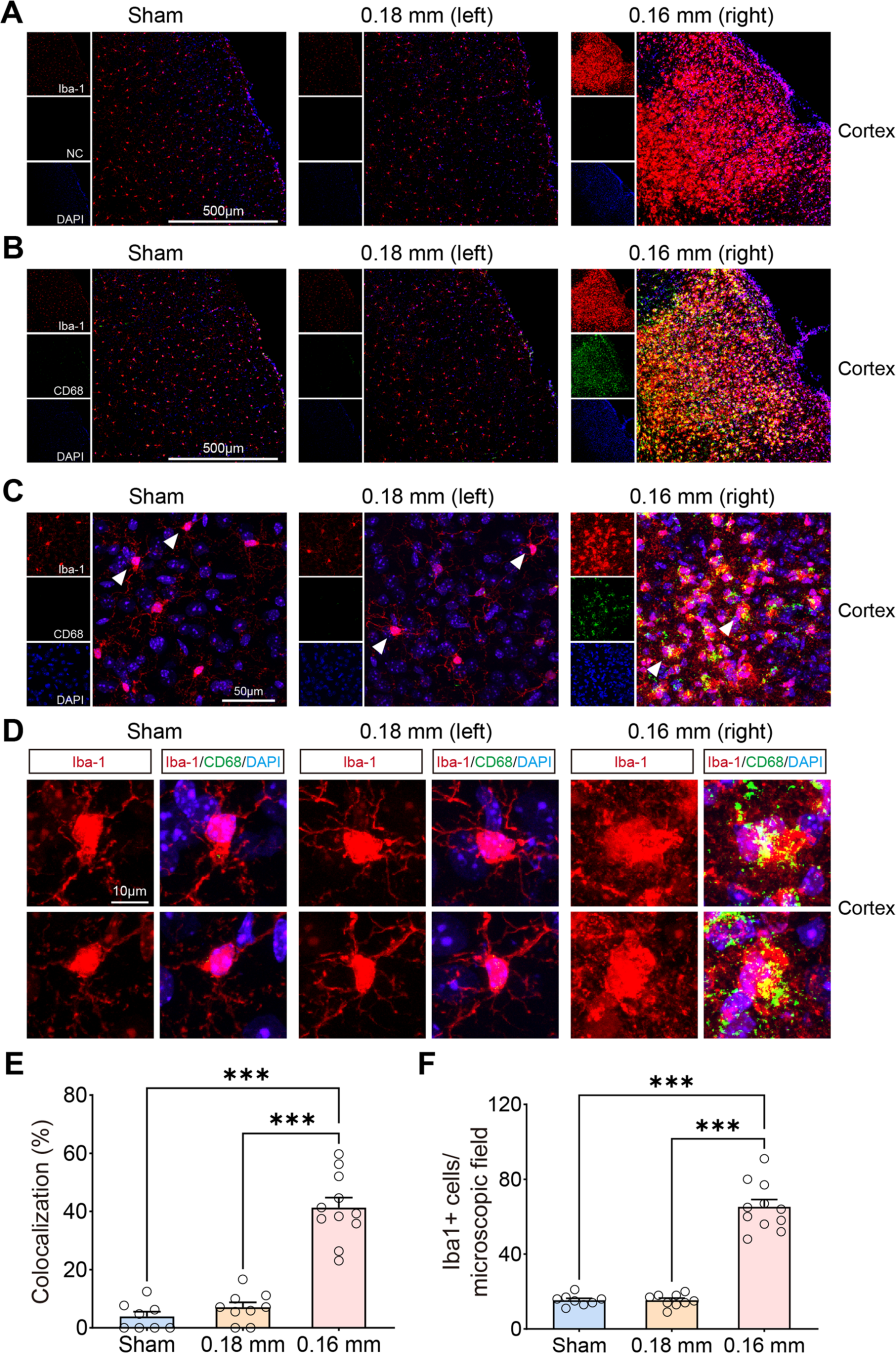
Activated microglia examined by CD68/Iba-1 double-staining after BCAS hypoperfusion. **A–B** Representative confocal images in the cerebral cortex (10 ×). Sections were stained antibodies against Iba-1 and CD68. NC, negative control without the CD68 primary antibody. The data rule out the background caused by the secondary antibody, as evidenced by NC. **C–D** Representative images at higher resolution of CD68/Iba-1 immunofluorescence showing microglial cells positive for CD68 (arrowheads) in BCAS mice. *N* = 6/group. **E–F** Quantitative analysis revealed that the number of colocalized pixels and the number of Iba1-positive cells were significantly higher on the 0.16 mm stenosis side of BCAS mice than other groups. One-way ANOVA test. Error bars represent mean ± SEM. ***p < 0.001

Here, we investigated activated microglia using CD68/Iba-1 double-staining after BCAS hypoperfusion, as microglial activation is a key element in initiating and perpetuating inflammatory responses to ischemia. Results showed that the ratio of CD68+Iba1+/Iba1+ cells and the number of Iba1+ cells was significantly higher on the 0.16 mm stenosis side of BCAS mice than other groups (Fig. 3); indicated part of activated microglia/macrophage displayed phagocytosis phenotypes following BCAS hypoperfusion.

### Cortex-specific Up-regulated Genes Induced by BCAS Hypoperfusion were Enriched in a Specific Cell-type in the Brain

**Fig. 4:**
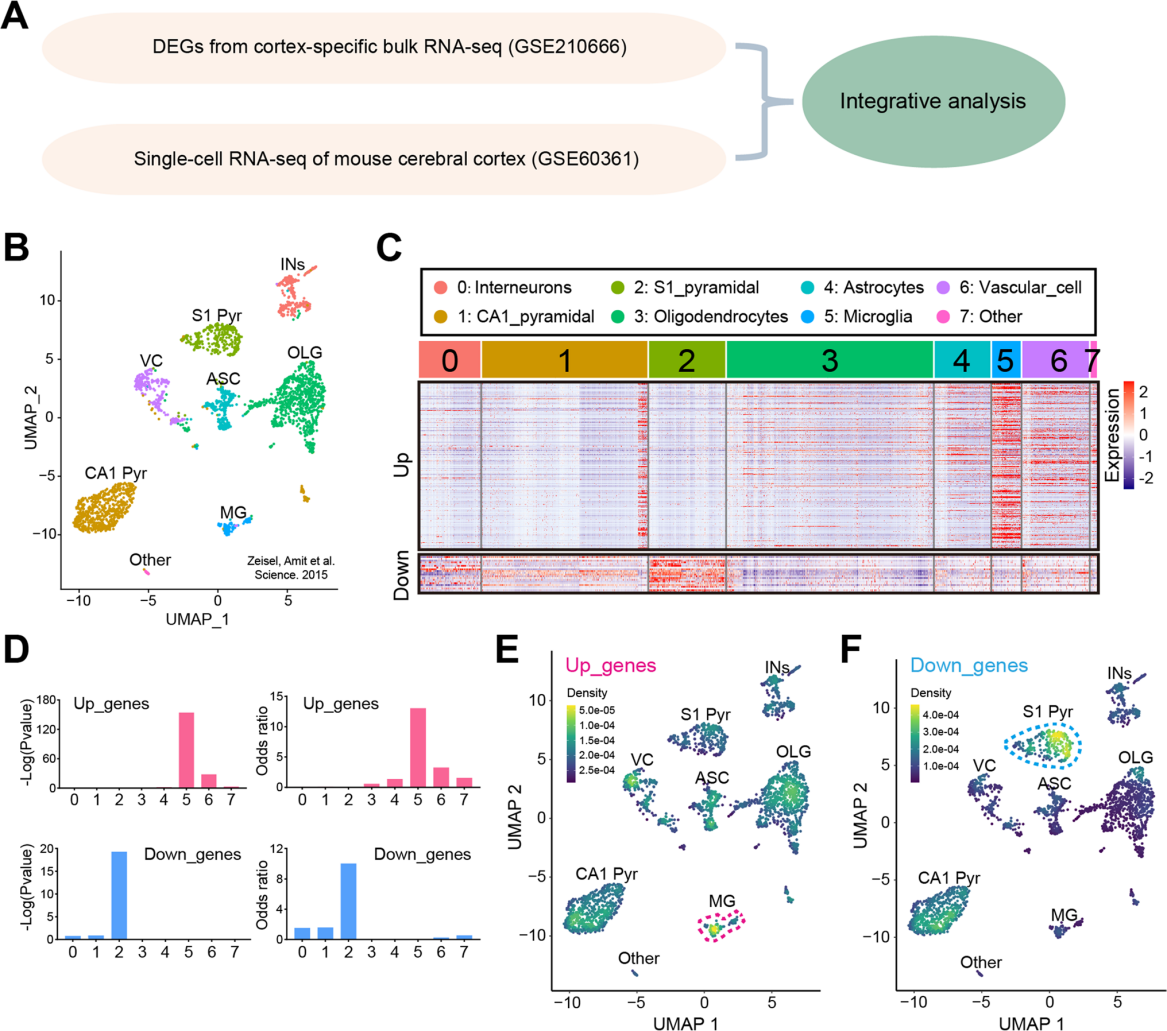
Distinct enrichment of BCAS hypoperfusion-induced DEGs in brain cell types. **A** Schematic diagram of the analysis strategy. **B** The UMAP map visualizing clustering of single cells colored by cell types using published single-cell RNA-seq data (Zeisel, A. et al., 2015, Science). **C** The individual gene expression level of DEGs induced by BCAS hypoperfusion in each cell of the published single-cell RNA-seq dataset (GSE60361) in Seurat’s DoHeatmap. **D** The bar plots show the gene set enrichment score and odds ratio (95% CI) of up-regulated genes (Up_genes) and down-regulated genes (Down_genes) in each cell type. **E–F** Density scatterplots showing cell-type enrichment for “Up_genes” and “Down_genes” using *UCell* R package (https://github.com/chuiqin/irGSEA). Visualized genes are DEGs from the cortex-specific RNA-seq dataset in our prior study. Notice the enrichment of up-regulated genes in cluster 5 of “MG” and down-regulated genes in cluster 2 of “S1 Pyr.” Ins interneurons, CA1 Pyr CA1 Pyramidal, S1 Pyr S1 Pyramidal, OLG oligodendrocytes, ASC astrocytes, MG microglia, VC vascular cells, Other other cell types

Previous studies have revealed different cell types in the mouse brain using single-cell RNA-seq under normal or pathological conditions [27–29]. The schematic diagram of the analysis strategy is shown in Fig. 4A. As shown in Fig. 4B and Supplementary Fig. 2, eight transcriptionally distinct clusters were identified via the detection of known cell type markers using a published scRNA-seq dataset (GSE60361) of brain cells [27]. The eight major classes of cells included “Interneurons” (cluster 0), “CA1 Pyramidal” (cluster 1), “S1 Pyramidal” (cluster 2), “Oligodendrocytes” (cluster 3), “Astrocytes” (cluster 4), and “Microglia” (cluster 5), “Vascular cells” (cluster 6), and “Other” (cluster 7). It should be added that vascular cells are composed of three cell types: vascular endothelial cells, vascular smooth muscle cells, and perivascular cells. Next, we visualized the expression of our cortex-specific DEGs in single-cell of this published scRNA-seq dataset. We found a distinct distribution pattern in “microglia” for the “BCAS-induced up-regulated genes” (Up_genes), and in “S1 Pyramidal” for the “BCAS-induced down-regulated genes” (Down_genes) (Fig. 4C). In addition, gene set enrichment analysis showed that the set of up-regulated genes was significantly enriched in “microglia” (*p*=1.02e−154, odds ratio 13.0). In contrast, the down-regulated gene set was enriched in “S1 Pyramidal” (*p*=5.21e−20, odds ratio 10.0), but not in “Interneurons” or “CA1 Pyramidal” (Fig. 4D). In addition, we did cell-type enrichment analysis using the *UCell* algorithm in irGSEA. As shown in Fig. 4E–F, a distinct “BCAS-induced up-regulated genes” distribution pattern in “microglia” was observed. We also noticed a unique enrichment for the “BCAS-induced down-regulated genes” in another “S1 Pyramidal” class. In summary, these results indicate that distinct cell types in the cortex responded to BCAS hypoperfusion differentially with the transcriptional activation occurring primarily in “microglia” as well as repression in “S1 Pyramidal.”

### The Up-regulation of IFN Signaling Molecule Expression Induced by BCAS Hypoperfusion is Related to Microglial Activation

**Fig. 5:**
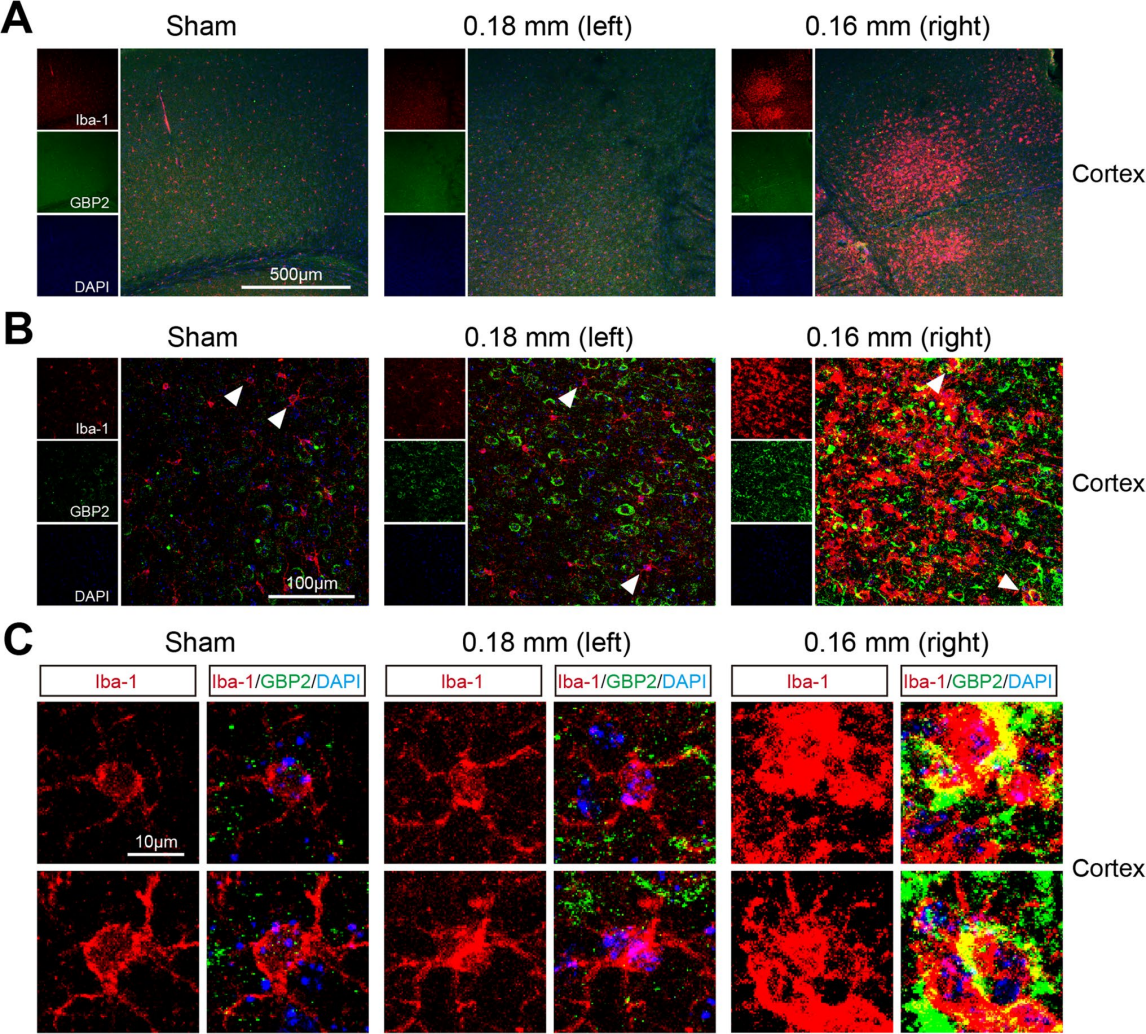
Altered phenotype of microglia was accompanied by elevated IFN-β signaling marker expression in the cerebral cortex after BCAS hypoperfusion. **A** Representative confocal immunofluorescence images marked by GBP2/Iba-1 after BCAS hypoperfusion (10 ×). **B** The confocal image of GBP2/Iba-1 co-staining of cortical region in both sham and BCAS groups (40 ×). **C** High-magnification fields (arrow areas) show colocalization of GBP2 (green) with Iba-1 (red)

In our previous research, gene set enrichment analysis (GSEA) revealed that BCAS hypoperfusion-induced up-regulated genes were significantly enriched in the pathways of IFN-regulated signaling along with neuroinflammation signaling. Also, our work revealed elevated expression of IFN-inducible protein in the cerebral cortex following BCAS hypoperfusion [15]. Based on the integrative analysis of our RNA-seq data and the published scRNA-seq dataset, it can be seen that the change of gene expression caused by BCAS hypoperfusion is closely associated with the activation of microglia. As the functional protein induced by IFNs, GBP2 plays an important role in response to IFN-β signaling. In this study, we performed double immunostaining of GBP2 and Iba-1 (microglia marker) to identify the expression and cellular localization of GBP2 following BCAS hypoperfusion. The results demonstrated that BCAS hypoperfusion enhanced the expression level of GBP2 in the cerebral cortex of the BCAS group, compared with the sham group (Supplementary Fig. 3). Representative confocal images of the cortices were taken, and colocalization of GBP2 with partially activated microglia was observed around the lesion site on the 0.16 mm stenosis side in the BCAS group (Fig. 5). Furthermore, microglial activation was accompanied by enhanced production of GBP2, and GBP2 was activated in microglia following BCAS hypoperfusion. These findings were significant, as they suggested that distinct cell types in the brain responded to BCAS hypoperfusion differentially and that up-regulated gene expression was related to IFN-I signaling in microglia.

### Alterations of Chromatin Accessibility in the Cerebral Cortex Following BCAS Hypoperfusion

**Fig. 6:**
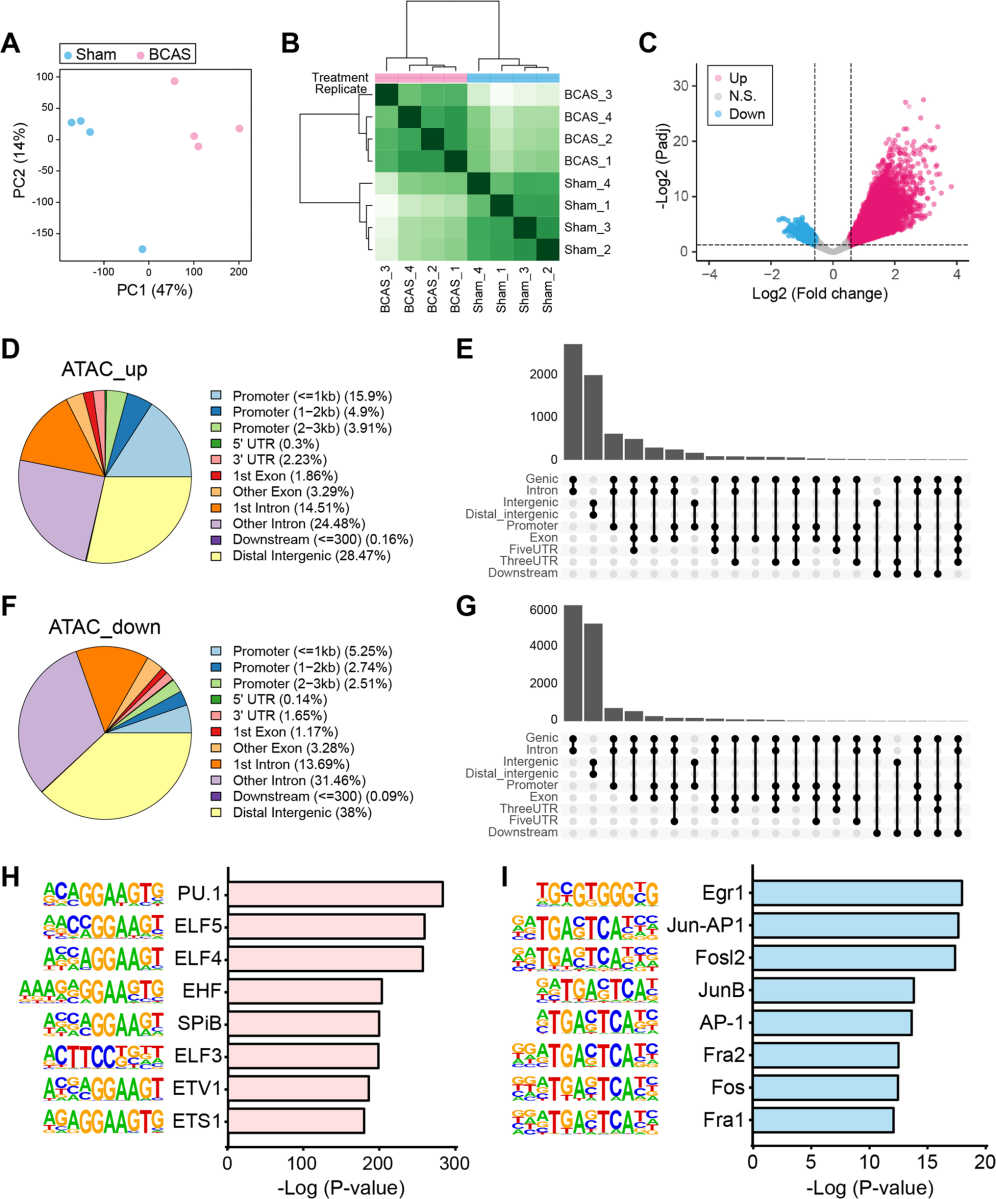
Cortex-specific alterations of chromatin accessibility following BCAS hypoperfusion. **A** PCA plot of ATAC-seq using isolated cerebral cortex from mice exposed to sham and BCAS operation. *N*=4 for each group. **B–C** The clustered heatmap and volcano plot show differential ATAC-seq signals in the cortex after BCAS hypoperfusion. Pink dot, up-regulated loci (Up); Blue dot, down-regulated loci (Down); Gray dot, non-significant loci. FDR < 0.05. **D** Genome annotations for up-regulated ATAC-seq loci. **E** Upset plots that show the overlap of accessible regions for up-regulated loci across genomic features. **F** Genome annotations for down-regulated ATAC-seq loci. **G** The overlap of accessible regions for down-regulated loci across genomic features. **H–I** Homer de novo motif prediction for up- and down-regulated loci

In our previous work, cortex-specific transcriptome profiling analysis was conducted on both sham and BCAS mice [15]. Based on our findings of gene expression changes in the current BCAS model, we hypothesized that these transcriptional changes were a result of chromatin accessibility. Recent emerging technologies make possible the combined investigation of epigenomics and transcriptomics using the same tissue. Therefore, in order to further study the mechanics underlying CCH-induced differential gene expression in the cortex, we performed ATAC-seq to determine the genome-wide dynamics of the chromatin accessibility of the cortex in the BCAS hypoperfusion model. Approximately 98% of paired-end reads mapped to the mouse reference genome and provided an average mapping depth of 40.5 million reads per sample (Supplementary Table 2). Principal component analysis (PCA) revealed a divergence of ATAC-seq signal between the sham and BCAS mice (Fig. 6A). Also, the heatmap shows a clear distinction between the two groups and the clustering trend within each group (Fig. 6B). Differential analysis identified significantly altered ATAC-seq loci (FDR <0.05) (Fig. 6C). Most of the loci, both up- and down-regulated, were annotated to the noncoding sequence regions: “ATAC_up,” 28.5% at distal intergenic, 24.7% at promoter, and 39.0% at intron; “ATAC_down,” 38.0% at distal intergenic, 10.5% at promoter, and 45.2% at intron (Fig. 6D–G). The distribution of differentially accessible regions (DARs) showed that almost half of these regions were present in genic and intron regions. It was also shown that the number of DARs in genic-intron was greater than in the intergenic regions. These changes in genomic distribution of chromatin accessibility after BCAS hypoperfusion may facilitate differential gene transcription through chromatin-level regulation. Homer motif search further identified multiple important TF binding motifs for the “ATAC_up” and “ATAC_down” loci (Fig. 6H–I). Notably, PU.1 (also known as SPI1) motif was the one most enriched in the “ATAC_up” loci, followed by ELF5 and ELF4.

### Validation of CCH-induced Cortex-specific Gene Expression and Chromatin Accessibility Changes by qRT-PCR

**Fig. 7:**
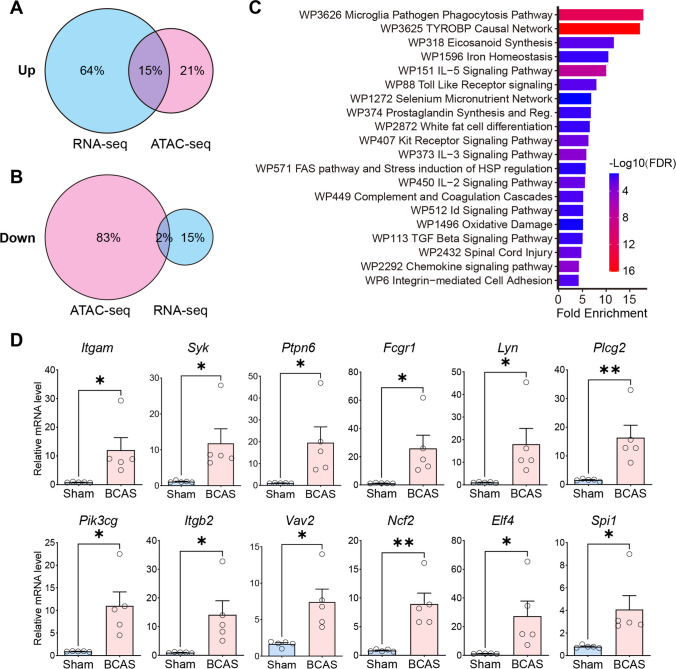
GO analysis of gene expression and chromatin accessibility changes after BCAS hypoperfusion. **A–B** Venn diagrams of up-regulated and down-regulated genes from integrative analysis of both RNA-seq and ATAC-seq data. The blue color represents the DEGs of RNA-seq analysis, and the red color represents the closest peak related genes of ATAC-seq analysis. **C** The GO functional enrichment analysis of up-regulated genes shared by RNA-seq and ATAC-seq data. **D** Validation of differential gene expression by quantitative RT-PCR on isolated nuclei from cerebral cortex of sham and BCAS mice. Expression of mRNA indicated as relative expression compared to the housekeeping gene *Gapdh*. Up-regulated genes involved in “Microglia pathogen phagocytosis pathway” *(Itgam*, *Syk*, *Ptpn6*, *Fcgr1*, *Lyn*, *Plcg2*, *Pik3cg*, *Itgb2*, *Vav2*, *Ncf2*) and “Representative transcription factors binding motifs for the ATAC_up loci” (*Spi1*, *Elf4*). Data are expressed as mean ± SEM. Unpaired *t* test (two-tailed), *p ˂ 0.05, **p ˂ 0.01 versus sham. *N* = 5 mice for each group

In order to obtain the functional information on the above peak, the peak-related genes were identified, annotated, and enriched. The joint analysis of differential peak-related genes and DEGs in RNA-seq showed that the shared DEGs were mainly up-regulated (Fig. 7A–B), and the differentially up-regulated genes between the two accounted for 15% (a total of 429 genes). The above differential genes were subjected to the GO knowledgebase for function enrichment analysis (Fig. 7C). Functional annotation of the differential up-regulated genes after BCAS hypoperfusion showed enrichment of molecular functions involved in “microglia pathogen phagocytosis pathway.” To detect the expression of genes highly enriched in the microglia phagocytosis-related signaling pathway, including *Itgam*, *Syk*, *Ptpn6*, *Fcgr1*, *Lyn*, *Plcg2*, *Pik3cg*, *Itgb2*, *Vav2*, and *Ncf2*, qRT-PCR analysis was performed on isolated nuclei from the cerebral cortices of sham and BCAS mice. Compared with the sham group, BCAS hypoperfusion induced higher mRNA levels related to these genes (Fig. 7D). Furthermore, the mRNA expression levels of transcription factors ELF4 and SPI1 (also known as PU.1) annotated by motif analysis were significantly increased in the BCAS group (Fig. 7D). These results were consistent with the findings of our ATAC-seq analysis.

### Verification of the Upstream Core Regulatory Factor PU.1 Induced by BCAS Hypoperfusion

**Fig. 8:**
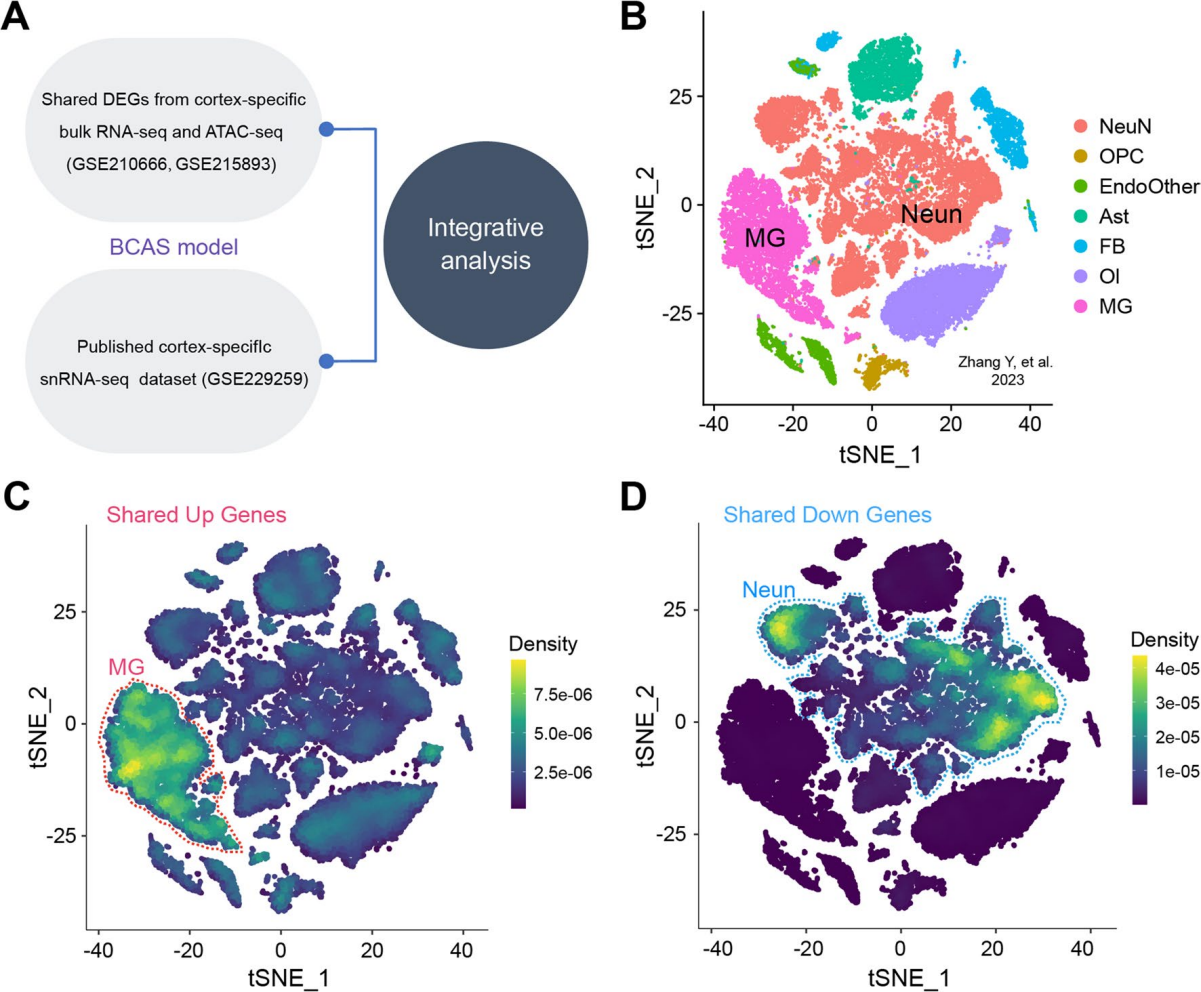
Shared up-regulated DEGs from cortex-specific RNA-seq and ATAC-seq were enriched in microglia. **A** The analysis strategy. **B** T-distributed stochastic neighbor embedding (t-SNE) plot shows all single-nuclei colored by cell types using published data (Zhang, Y. et al., 2023, J Neuroinflammation). **C–D** t-SNE plots show the enrichment of snRNA-seq for shared “Up_genes” and “Down_genes” by using irGSEA analysis with the *UCell* algorithm, respectively. Visualized genes are DEGs from cortex-specific RNA-seq and ATAC-seq dataset in our study. Notice the enrichment of up-regulated genes in cluster of “MG” and down-regulated genes in cluster of “Neun.” Neun neuron, MG microglia, Ast astrocyte, OI oligodendrocyte, OPC oligodendrocyte precursor cell, FB fibroblast, EndoOther endothelial cell and other cell

**Fig. 9:**
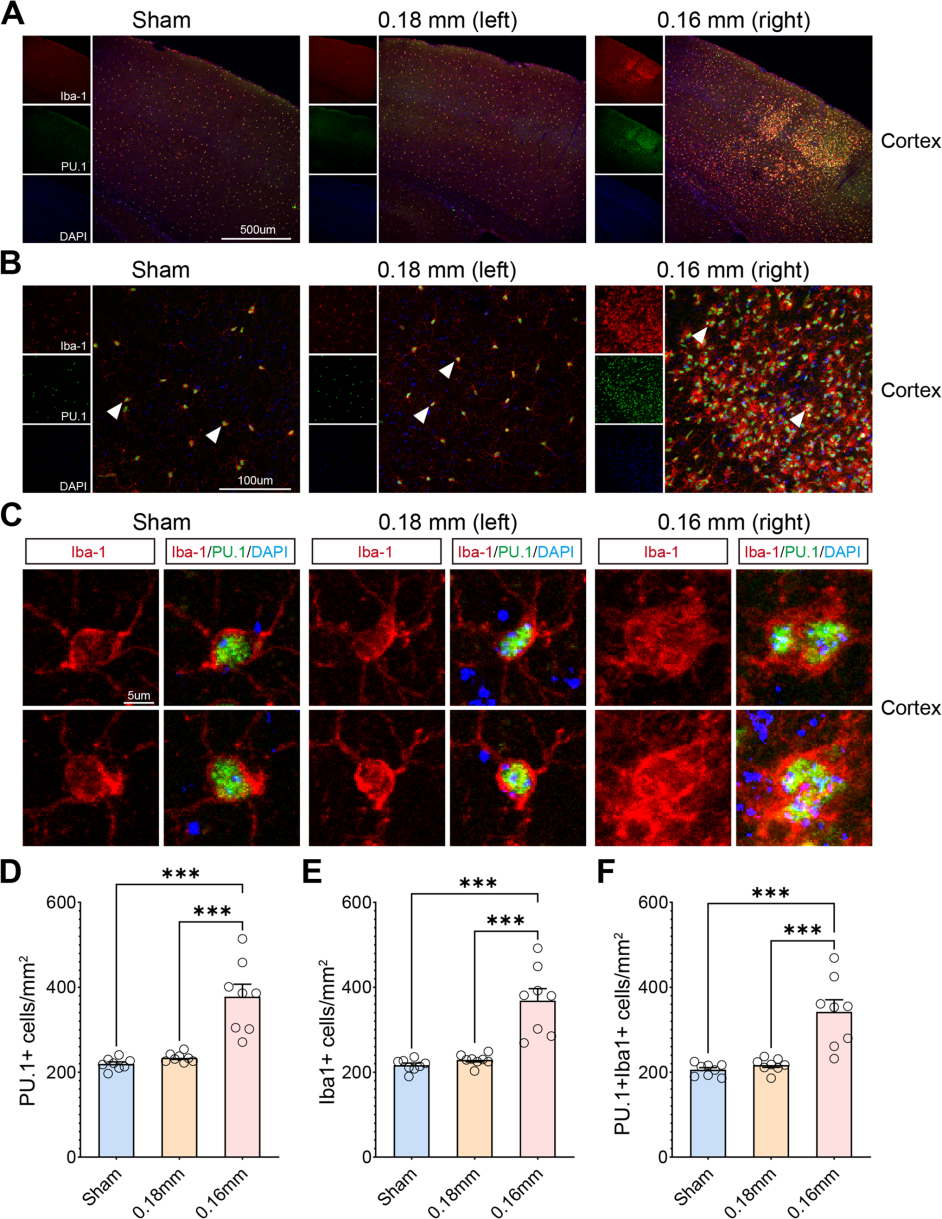
Increased number and activated morphology in Iba1+PU.1+microglia in the cerebral cortex of BCAS mice. **A** Representative confocal immunofluorescence images of PU.1/Iba-1 co-staining of the cerebral cortex in sham and BCAS groups (10 ×). **B** Representative confocal images of PU.1/Iba-1 co-staining of cortical region in both sham and BCAS groups (40 ×). *N* = 8 mice/group. **C** High-magnification fields (arrow areas) show colocalization of PU.1 (green) with Iba-1 (red). **D–F** Quantification of PU.1+ cells/mm^2^, Iba1+ cells/mm^2^, and PU.1+Iba1+ cells/mm^2^, respectively. Notice the number of PU.1+Iba1+ cells was significantly higher on the 0.16 mm stenosis side of BCAS mice than other groups. One-way ANOVA test. Error bars represent mean ± SEM. ***p < 0.001

From the results of the RNA-seq and ATAC-seq analysis, the chromatin accessibility changes induced by BCAS hypoperfusion were shown to be closely related to microglial activation, and it was shown that PU.1, encoded by *Spi1*, plays a significant role in cortical injury and microglial activation caused by CCH. Recent studies have shown that researchers have performed cortex-specific single-nuclei RNA sequencing (snRNA-seq) following BCAS hypoperfusion (GSE229259) [30]. Given this, we considered further joint analysis with our dataset. The analysis strategy is shown in Fig. 8A. After combining our cortex-specific bulk RNA-seq and ATAC-seq analysis (GSE210666, GSE215893), we analyzed the shared DEGs between the two, then we conducted cell type enrichment analysis on the shared DEGs with the snRNA-seq dataset. Interestingly, we found that shared up-regulated DEGs from cortex-specific RNA-seq and ATAC-seq were enriched in microglia as well as down-regulated DEGs were enriched in neuron (Fig. 8B–D). Immunofluorescent staining revealed that cerebral hypoperfusion resulted in increased expression of PU.1 in the cerebral cortices on the 0.16 mm stenosis side of BCAS mice than other groups (Supplementary Fig. 4). In addition, the laser scanning confocal images showed colocalization of PU.1 with Iba-1 (Fig. 9A–C). Quantitative analysis demonstrated that the number of PU.1+Iba1+ cells was significantly higher on the 0.16 mm stenosis side of BCAS mice than other groups (Fig. 9D–F). More importantly, as shown in Supplementary Table 6, upstream analysis was conducted on the shared DEGs between cortex-specific bulk RNA-seq and ATAC-seq. Results revealed that both PU.1 and interferon regulatory factor-1 (IRF1) can regulate the DEGs induced by BCAS hypoperfusion as well as the ability of PU.1 to bind with promoter regions of the shared DEGs. These findings are significant, as they indicate that interaction between microglial PU.1 function and IFN response may regulate a sequence of immune inflammatory events after CCH.

## Discussion

In this study, we investigated and verified the cellular and molecular mechanisms of chronic hypoperfusion-induced brain injury from the transcriptome level. Our research revealed that the up-regulated genes induced by BCAS hypoperfusion were specifically enriched in microglia by integration with scRNA-seq data, and that IFN-β signaling is involved in regulating microglial activation induced by CCH. Integrated analysis of ATAC-seq and RNA-seq showed that microglia pathogen phagocytosis response is a critical factor in CCH-induced brain injury. Analysis of TFs identified a key TF, PU.1 (encoded by *Spi1* gene), which is the upstream core regulator of IFN-β signaling in microglia activation caused by BCAS hypoperfusion.

Numerous rodent models have been developed that mimic features of VCI [5]. Because of the general, or at least local, reduction in CBF in elderly patients with VCI, a CCH model has been established in rats, gerbils, and mice to characterize these persistent cerebral ischemia features in humans [31–33]. Notably, the BCAS mouse model has been evaluated and determined to be one of the most germane rodent models of CCH, and various researches have used the BCAS animal model to investigate potential therapeutics for VCI [34, 35]. Studies have shown that after BCAS surgery with bilateral 0.16 mm inner diameter microcoils, the CBF decreased to (51.4 ± 11.5)% at 2 hours after operation, and the mortality rate was about 75% at 14 days after the operation. Although this procedure can cause gray matter lesions, bilateral 0.16 mm stenosis lowers survival rates in mice, which limits its wide application [36]. In addition, BCAS model with bilateral 0.18 mm microcoils reduced the CBF to (67.3 ± 18.5) % at 2 hours after the operation, and the mortality rate at 14 days post-surgery was about 15%. Although this procedure has a high survival rate, the bilateral 0.18 mm BCAS model causes only white matter lesions and does not cause significant gray matter damage [37–39]. Subsequently, researchers have used the modified BCAS model, that is, the 0.16/0.18 mm BCAS model, which can cause asymmetric ischemia in the left and right hemispheres [40]. The postoperative mortality rate of this model is low (close to 20%), and asymmetric ischemia is a better representation of the actual state of clinical disease [20]. The researchers also found that the 0.16/0.18 mm BCAS model can cause diffuse infarction on the 0.16 mm stenosis side, cause clearly evident histological damage to gray matter with glial activation, and more accurately simulate the pathological process of CCH in humans. In our research, the results of postoperative behavioral analysis, histological staining, and immunofluorescence staining all verified this model. By using 0.16/0.18 mm microcoils to induce BCAS hypoperfusion, chronic cerebral ischemia can be more accurately simulated, and a stable and effective hypoperfusion mouse model can be established. In our study, microinfarction or mild to moderate infarction was observed histologically at 3 weeks after BCAS hypoperfusion, and infarction-like lesions were scattered throughout the whole brain, which was consistent with the results of previous studies [15, 40]. Furthermore, several previous studies have shown that the activation of microglia and the release of inflammatory cytokines reaches a peak in the middle and late stage of ischemic injury, and then decreases gradually [41]. Since clearly evident brain injury and microglial activation were observed 3 weeks after BCAS operation, we chose this point in time for subsequent experiments in order to explore the important cellular and molecular signaling pathways and key regulatory factors associated with CCH.

One of the most important aspects of this study was the investigation of IFN-β signaling in regulating microglial activation induced by CCH. While the significant role of type I interferon (IFN-I) signaling in fighting central nervous system infections is well recognized [42, 43], the role and underlying cellular mechanisms in CCH are not yet well understood. Interestingly, the activation of the IFN-I signaling pathway has been determined as a factor in a variety of animal models of brain disorders [44, 45]. Several studies have shown that IFN-I can promote the release of pro-inflammatory factors and participate in regulating brain injury, thereby affecting the interaction between different cell types in the brain [14, 46]. In a recent study, microglia were taken from the brain tissue of a typical traumatic brain injury (TBI) mouse model, RNA-seq was performed, and the researchers found that the gene sets of “IFN-I signaling” and “MHC class I antigen presentation pathway” were significantly enriched after TBI [14]. This shows that IFN-I response participates in the regulation of TBI-induced phenotypic changes in microglia. Also, microglia-specific transcriptional changes indicated the importance of cell-type specific analysis of brain cells after brain injury and can help to identify new targeted therapies. In addition, other studies have found that IFITM3 (IFN-induced protein) can play a similar role to γ-secretase regulatory protein through neuroinflammatory mechanics [47], and IFITM3 can also be considered a new molecular target of IFN-β signaling involved in regulating microglia reactivity to various inflammatory responses [48]. Research has shown that microglial bridging integrator 1 (BIN1) function regulates IFN-I response and participates in regulating disease-associated microglia (DAM) phenotypes [49]. Our study also verified the importance of IFN-β signaling molecules in reactive microglia induced by BCAS hypoperfusion, which is consistent with previous studies.

Several molecules in the guanylate-binding protein (GBP) family are induced by IFN signals, including GBP2, which is considered to be a marker of IFN signaling response [50]. A previous study has shown colocalization of GBP2 and Iba-1 in the cerebral cortex by immunofluorescence staining in a rat model of TBI-induced brain injury, and that the increased expression level of GBP2 was mainly related to activated microglia [51]. These results suggest that GBP2 may play an important role in the activation of microglia after brain injury. In our study, through the integration analysis of bulk RNA-seq data and scRNA-seq data, it was found that CCH-induced up-regulated genes were specifically enriched in microglia, and laser scanning confocal observation also confirmed the colocalization of GBP2 with activated microglia. We speculate that IFN signaling and response participate in regulating microglial activation induced by CCH.

The growth and differentiation of microglia are regulated by a variety of factors, including colony-stimulating factor 1 receptor (CSF1R), PU.1, and IRF8 [52, 53]. PU.1, a member of the ETS family of TFs, is dynamically expressed in neutrophils, mast cells, and microglia/macrophages, and can regulate the proliferation and differentiation of hematopoietic stem cells and play an important role in regulating immune response [54]. Recent research has reported that the transcription factor PU.1 is critical for viability and function of human brain microglia [55]. Also, PU. 1 regulates the expression of several microglial genes, and lower PU.1 expression in myeloid cells could delay aged-associated neurodegenerative diseases onset [49]. Therefore, as a significant regulator in the development and proliferation of microglia, the expression of PU.1 protein can be affected by changes in the functional phenotype of microglia. Interestingly, in our study, immunofluorescence staining showed that the expression level of PU.1 was greatly increased and colocated with Iba-1 induced by BCAS hypoperfusion. Therefore, PU.1, as a core TF, participates in regulating microglial activation induced by CCH. These results are of great significance, as they indicate that PU.1 may be the upstream core regulator in CCH-induced microglial activation. The regulatory relationship between PU.1 and IFN signal is not yet definitive based on current research results. However, our previous work confirmed the role of the IFN signal in regulating BCAS hypoperfusion state [15]. Up-regulated gene expression caused by BCAS hypoperfusion was closely related to IFN signaling in microglia (Figs. 4 and 5). Also, our multi-omics analysis demonstrated that PU.1 plays a central regulatory role in BCAS hypoperfusion-induced microglial activation. More importantly, studies have shown that there are co-regulatory effects between transcription factors PU.1, interferon regulatory factor 1 (IRF-1) and interferon consensus sequence-binding protein (ICSBP). Serine 148-phosphorylated PU.1 increases gp91(phox) expression by interacting with both IRF-1 and ICSBP [56, 57]. Thus, we speculate that interaction between microglial PU.1 function and IFN-I response may regulate a sequence of immune inflammatory events after BCAS hypoperfusion. In short, our study investigated cortex-specific gene expression alterations induced by BCAS hypoperfusion as well as the regulatory mechanics involved in these changes through multi-omics integrated analysis. It will be of great significance to investigate how these IFN-mediated neuroimmune responses participate in the detailed molecular mechanisms of microglial activation caused by chronic cerebral ischemic injury.

In summary, the effect of chromatin accessibility on gene expression was verified through integration analysis of RNA-seq and ATAC-seq, and the molecular mechanism of gene expression regulated by the key regulator was researched. Our study reveals the complex regulatory mechanics of IFN signaling-mediated neuroimmune processes following CCH at the levels of chromatin, TFs, and genes, and shows that PU.1 may be the core of this regulator network. These results provide a specific target for the early intervention of neuroimmune response after CCH.

## Conclusions

We investigated the gene expression and transcriptional regulation alterations profile underlying CCH using BCAS model. The results indicated distinct cell types in the cortex responded to BCAS hypoperfusion differentially with the transcriptional activation occurring primarily in “microglia” as well as repression in “S1 Pyramidal.” Up-regulated gene expression was related to IFN-I signaling in microglia. It is novel to compare the distinct enrichment of DEGs in brain cell types following CCH.

### Limitations of the Study

Although our study provides a new dataset of cortex-specific chromatin and transcriptomic profiling after BCAS hypoperfusion in mice, it has some limitations. First of all, this study demonstrated that the changes of cortex-specific transcriptome are closely related to microglia, so the cell-type specific transcriptome alterations induced by BCAS hypoperfusion merit further research. Secondly, future studies are needed to clarify whether IFN-β signaling molecules, such as IFITM3, have the ability to regulate the microglia phenotype following CCH-induced brain injury. In the future, we plan to further experiment by blocking or knocking out the key effector molecules of IFN-β signaling, and it will be interesting to observe the effects of these alterations on microglia, neuron phenotype, and animal behavior function in vivo and in vitro. The follow-up work is expected to explore the more complex mechanics of BCAS hypoperfusion, in order to provide insights regarding the development of related drug targets and clinical treatment.

### Supplementary information

ESM 1

ESM 2

ESM 3

ESM 4

ESM 5

ESM 6

ESM 7

## Data Availability

The datasets provided in this study can be found in the online repository. The names of the repository/repositories and accession number(s) can be found below: https://www.ncbi.nlm.nih.gov/geo/query/acc.cgi?acc=GSE210666. https://www.ncbi.nlm.nih.gov/geo/query/acc.cgi?acc=GSE215893.

## Abbreviations

CCH: Chronic cerebral hypoperfusion
VCI: Vascular cognitive impairment
BCAS: Bilateral common carotid artery stenosis
RNA-seq: RNA-sequencing
DEGs: Differentially expressed genes
scRNA-seq: Single-cell RNA sequencing
ATAC-seq: The assay for transposase-accessible chromatin with high-throughput sequencing
IFN-I: Type I interferon
GBP2: Guanylate-binding protein 2
DARs: Differentially accessible regions
CBF: Cerebral blood flow
TFs: Transcription factors
CCA: Common carotid arteries
