# Supplementary material for: Integrated Analysis of Chromatin and Transcriptomic Profiling Identifies PU.1 as a Core Regulatory Factor in Microglial Activation Induced by Chronic Cerebral Hypoperfusion
Source: Mol Neurobiol. 2023 Nov 2;61(5):2569–89. doi: 10.1007/s12035-023-03734-9 (PMC11043206; doi:10.1007/s12035-023-03734-9)
Supplement: Supplementary file 1 — ESM 1 [file 12035_2023_3734_MOESM1_ESM.pdf]

Supplementary Fig. 1. Histological changes in different sections 3 weeks after BCAS-hypoperfusion. Bar plots for "Percentage of infarct area" of each section in BCAS mice. Data are expressed as mean ± SEM. Paired t test (two-tailed), **p* < 0.05 versus 0.18 mm side, n=10 mice for each group.

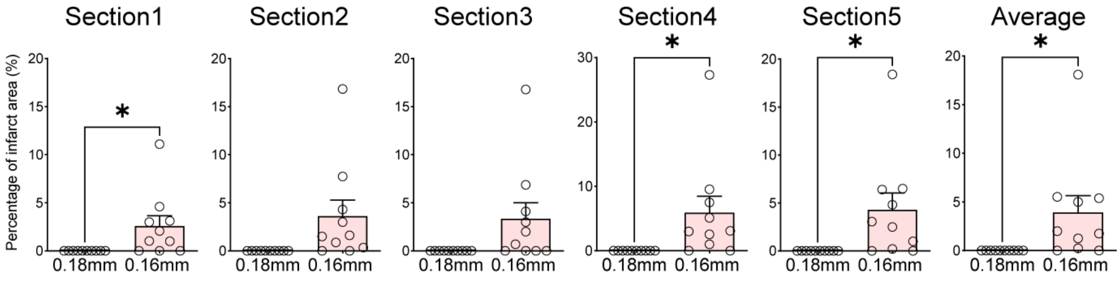

Supplementary Fig. 2. Cell clustering and visualization using the published scRNA-seq dataset (Zeisel, A. et al., 2015, Science). (**A**) The UMAP map visualizing the expression of marker genes in each cell population. (**B**) The UMAP map of the expression and distribution of marker genes in each cell population.

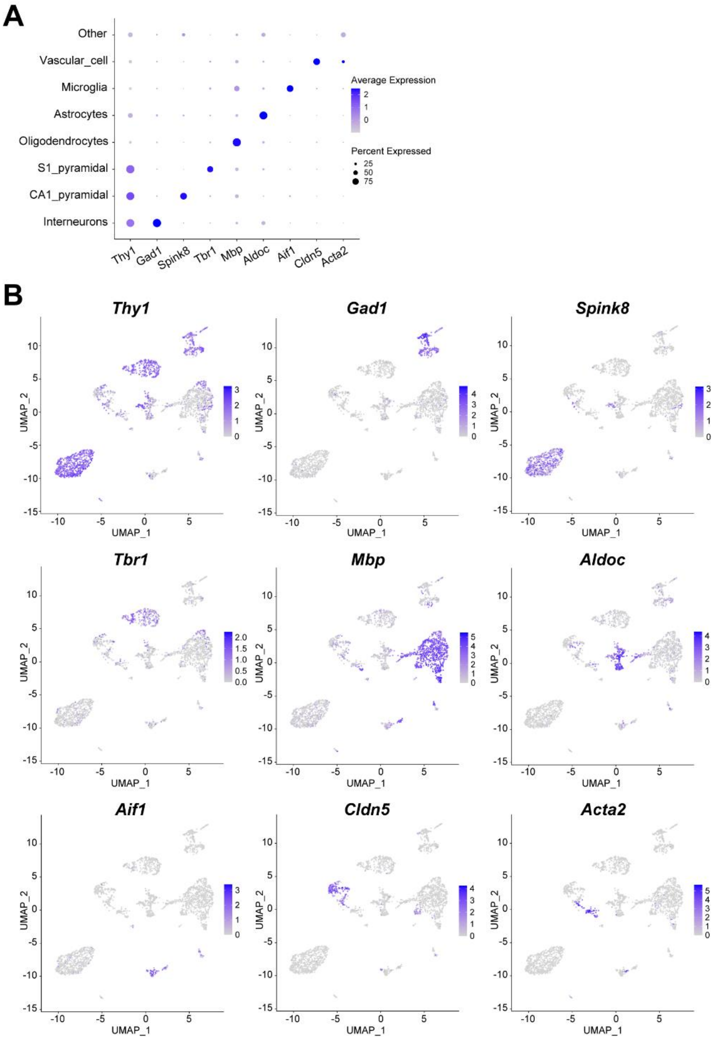

Supplementary Fig. 3. Immunofluorescence staining showed the expression of GBP2/Iba-1 in the cerebral cortex. (**A**) Representative brain slices labeled with Iba-1 and GBP2 in both sham and BCAS groups. (**B**) Representative immunofluorescence staining images of Iba-1/GBP2 in the cerebral cortex. (**C**) The quantified results of GBP2 expression level. One-way ANOVA, Bonferroni's multiple comparisons test, ***$p < 0.001$, n=8 mice for each group.

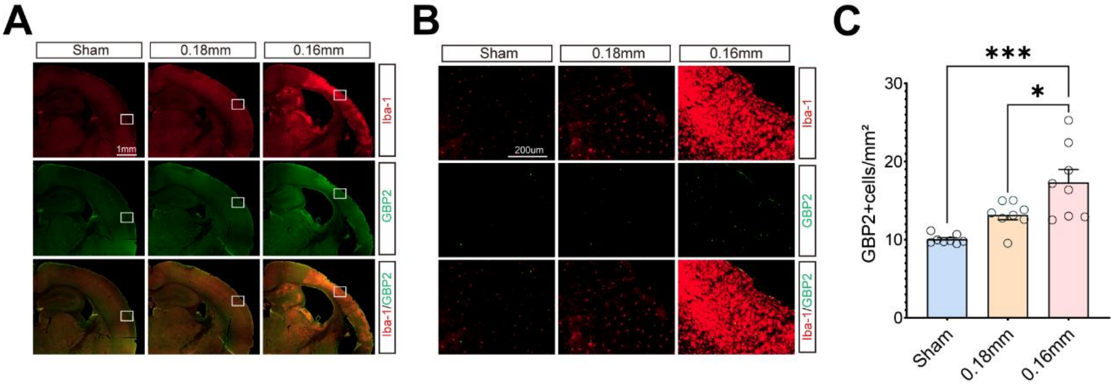

Supplementary Fig. 4. Microglial phenotypic change was examined by Iba-1/PU.1 double-staining following BCAS-hypoperfusion. (**A**) Representative immunofluorescence staining images labeled with Iba-1 and PU.1 in both sham and BCAS groups. (**B**) Representative images of Iba-1/GBP2 double-staining in the cerebral cortex.

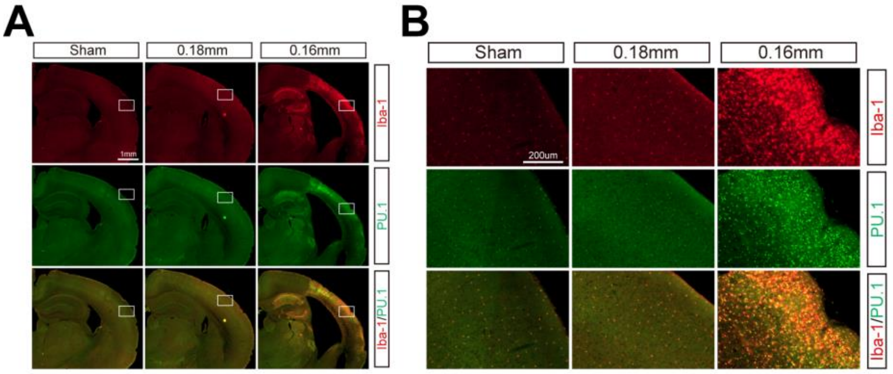

Supplementary Table 1. Primer sequences used for qRT-PCR.

Supplementary Table 2. Quality control for ATAC-seq data.

Supplementary Table 3. DARs obtained by ATAC-seq.

Supplementary Table 4. DEGs obtained by RNA-seq.

Supplementary Table 5. Shared DEGs for RNA-seq and ATAC-seq.

Supplementary Table 6. Enriched motif in promoter related to shared DEGs.
